# The ‘Public’ in Public Involvement: A Call to Centre Frontline Staff Voices in Health Workforce Research

**DOI:** 10.1111/hex.70616

**Published:** 2026-03-26

**Authors:** Yingxi Zhao, Justin Aunger, Hilary Garrett, Paul Hewitson, Alun Davies, Manish Pareek, Sassy Molyneux

**Affiliations:** ^1^ University of Oxford Nuffield Department of Medicine Oxford UK; ^2^ NIHR Midlands Patient Safety Research Collaboration University of Birmingham Birmingham UK; ^3^ Department of Applied Health Sciences, College of Medicine and Health University of Birmingham Birmingham UK; ^4^ Public Contributor National Institute for Health and Care Research and Health Data Research UK Lancaster UK; ^5^ Honorary Researcher Lancaster University Lancaster UK; ^6^ University of Oxford Nuffield Department of Population Health Oxford UK; ^7^ The Global Health network University of Oxford Oxford UK; ^8^ Division of Public Health and Epidemiology University of Leicester Leicester UK; ^9^ KEMRI‐Wellcome Trust Research Programme Kilifi Kenya

**Keywords:** co‐production, community engagement, health services research, public participation

## Abstract

Public involvement, or patient and public involvement and engagement (PPIE), is widely embedded in health and care research and is valued for its moral, ethical, and methodological contributions. However, its application in workforce research raises important questions about the voices that might be missing from the ‘public’ when research's primary object of inquiry is healthcare staff, organisations, and systems, rather than patients and service users. This viewpoint explores tensions arising from the extension of standardised public involvement requirements, which were originally developed for research where patients and service users are the primary target of focus, into workforce‐focused studies. We argue that while patient and public perspectives remain crucial, the lived experience and voices of frontline staff are often under‐recognised in existing involvement frameworks, including those used in NIHR domestic programmes, as they are not fully accommodated within either public involvement or stakeholder engagement activities. We therefore call for greater clarity, flexibility, and, where appropriate, expansion in how public involvement is conceptualised and operationalised in workforce research, to better centre the voices of frontline staff. Rather than offering definitive solutions, we invite debate and future research on how public involvement can be made more meaningful, proportionate, and fit‐for‐purpose in health and care workforce research.

**Patient or Public Contribution:**

This viewpoint article is written by researchers in the health and care workforce, PPIE, and community engagement, and a public contributor with interest and experience of involvement in health and care workforce research. We drew on our respective perspectives and experience to shape the framing and arguments presented in this viewpoint.

Health and care systems worldwide face mounting workforce pressures, rising service demands, and repeated system shocks. A growing body of health and care research has come to focus on those who deliver care, examining issues such as staffing and retention, workforce organisation, workplace culture, and staff wellbeing. Rather than patients and service users as the primary object of inquiry, this research unpacks the often ‘behind‐the‐scenes’ conditions under which care is delivered, recognising the workforce as central to system performance, safety, and sustainability, especially post‐COVID‐19. For example, one‐third of the UK National Institute for Health and Care Research (NIHR)‐funded Health and Social Care Delivery Research's live studies now have a core focus on the health and care workforce, including projects such as safe staffing levels, recruitment and retention of general practitioners, and implementation of new workforce roles [1]. Workforce‐focused research of this kind also spans other NIHR domestic programmes in public health, social care, and policy research, reflecting a sustained and expanding interest in workforce‐related challenges across the health and care systems, as also evidenced by the recent NIHR highlight notice [2]. These studies, however, are typically governed by the same public involvement requirements that were originally developed for clinical and patient‐focused research, in which patients and service users are the primary focus of inquiry and the most direct beneficiaries of the research. This juxtaposition raises a fundamental but under‐examined question: *Who is the “public” in public involvement for health and care workforce research?*

## 1 The Ethical and Methodological Foundations of Public Involvement

Public involvement, or patient and public involvement and engagement (PPIE), is widely acknowledged and embedded within health and care research and policy. It is morally and ethically necessary, methodologically valuable, and now a routine expectation of publicly funded research. Public involvement is intended to ensure that research is conducted ‘with’ or ‘by’ patients or members of the public, rather than ‘about’ them (Box 1) [3]. It is morally and ethically grounded in the principle that people have a right to be involved in research that affects them. Methodologically and operationally, public involvement has been shown to improve study relevance and acceptability, help shape research questions and priorities, enhance study recruitment and retention, and support the uptake of findings, particularly when involvement is clearly purposive and embedded across the research lifecycle [4].

Box 1 **Current UK National Institute for Health and Care Research definition of public involvement [3].**

**Table:** 

*We define public involvement in research as research being carried out ‘with’ or ‘by’ members of the public rather than ‘to’, ‘about’ or ‘for’ them. When we use the term ‘public’, we are including:*
*patients and potential patients*
*carers and people who use health and social care services*
*people from organisations that represent people who use services*

Recognition of the intrinsic and instrumental value of public involvement has contributed to its requirements being increasingly formalised, standardised, and mandated within research governance frameworks. Funders such as the NIHR have played a central role in promoting public involvement (although notably, NIHR's global health programme has instead focused on ‘community engagement and involvement’). There are clear expectations for public involvement, such as at funding application stage to describe how public contributors will be involved, supported, and recognised within projects (such as co‐applicants), and guidance, reporting requirements, and support have been developed to strengthen the quality and visibility of involvement across publicly funded research. However, this increasing standardisation has also tended to narrow how public involvement is interpreted and operationalised within NIHR's domestic programmes, often prioritising forms of involvement centred on individual patients and service‐users only. Critics have noted that such formalisation can contribute to the emergence of an ‘involvement industry’ [4], and warn of the risk that public involvement becomes routinised as a ‘tick‐box’ or performative exercise, undertaken primarily to satisfy funding requirements. Conducting public involvement well is inherently challenging, requiring careful attention to meaningful and reciprocal involvement aligned with the purposes of research [5], including consideration of whose voices are involved and how well they reflect wider perspectives, as well as substantial time and effort from all involved.

## 2 Applying Public Involvement to Health and Care Workforce Research

Questions about who constitutes the ‘public’ for public involvement become particularly ambiguous in health and care workforce research, where the immediate focus and the group most directly affected are often the workforce itself. In most clinical and health services research, the ‘public’ or ‘service users’ are commonly patients, carers, and other service users with direct experience of the condition, intervention, or service under study, as well as members of the wider public. In workforce‐focused research, patients and the wider public may remain the ultimate beneficiaries of improved staff wellbeing, working conditions, and organisational culture through safer and higher quality care, but these benefits are usually indirect and realised downstream. This complicates assumptions about who should be involved, in what capacity, and for what purpose within workforce‐focused public involvement.

These challenges become clearer when considering the focus of many workforce studies. For example, studies examining the retention of doctors and nurses, particularly in the contexts of severe and persistent workforce shortages, depend on insights into the day‐to‐day clinical realities faced by frontline staff, such as high workloads, unfilled rota shifts, changes in skill mix and role substitution, and the cumulative strain these pressures place on staff decisions about their working hours and longer‐term ability to remain in these frontline roles. These experiences are largely ‘behind‐the‐scenes’ of care delivery and are not necessarily directly visible to patients and the wider public, yet they are central to how workforce changes, such as those aimed at improving retention, are designed, implemented, and sustained, ultimately shaping the quality and continuity of patient care.

## 3 Frontline Staff Voices: Neither Patients and the Public, Nor Stakeholders?

Frontline health and care staff possess experiential and contextual knowledge grounded in the lived experience of the very systems, roles, and conditions under study. This knowledge is crucial to understanding how services function and how research findings can be implemented in practice. While their participation as research participants is essential for generating empirical data, this is distinct from recognising staff, particularly those in frontline roles, as partners in shaping research questions, design, interpretation and dissemination.

Such involvement also differs from engaging professional groups through stakeholder engagement, such as advisory groups. These mechanisms often operate at a strategic or representative level of the system, and may not fully capture the realities of frontline work. They are also not always resourced or structured in the same way as public involvement to support the same depth of influence across the research lifecycle, including research priorities, design, analysis, and dissemination.

**FIGURE 1:**
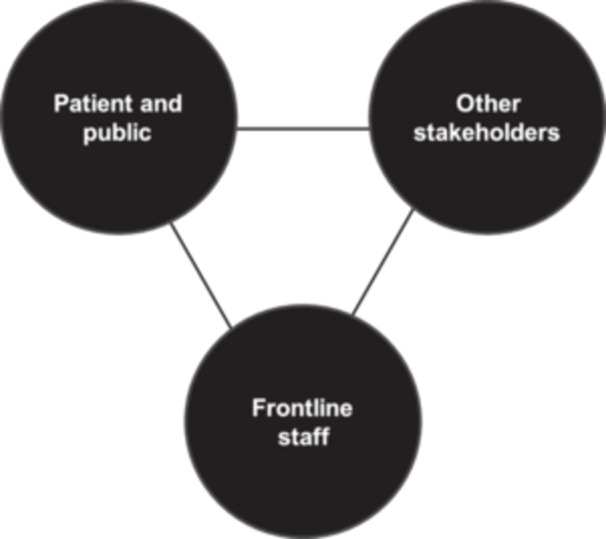
Distinct perspectives relevant to health and care workforce research.

Taken together, these distinctions point to three analytically distinct perspectives relevant to workforce research, that is, patients and the public, frontline staff, and other stakeholders as summarised in Figure 1. Frontline staff voices often fall between established PPIE and stakeholder categories, resulting in limited opportunities for meaningful involvement in shaping workforce research.

As a result, there is a risk that frontline staff perspectives and voices are neither recognised as public involvement within dominant involvement frameworks, nor fully captured by stakeholder mechanisms that disproportionately reflect managerial, organisational, or professional viewpoints rather than the direct experiences of frontline staff. Excluding or side‐lining the perspectives of frontline staff risks limiting the relevance, feasibility, and impact of workforce research, while also overlooking opportunities to support staff voice, agency, and participation in shaping research that directly concerns their working lives [6, 7]. Highly standardised or restrictive public involvement approaches that focus exclusively on patients and the general public, without involving frontline staff, may also lead to misalignment between the purpose of research and the forms of involvement activities and suboptimal use of patients' time and efforts within workforce‐focused studies. Further consequences may be the dilution of patient involvement in research contexts where patients should play a more central and influential role, and inefficient use of publicly funded research resources without delivering corresponding benefits.

## 4 Rethinking Involvement and Engagement for Workforce Research

We are not arguing against involving patients and the public in workforce research. Patient and public perspectives remain important in many service delivery and workforce studies, not only in relation to patient safety and continuity of care, but also in shaping priorities around access to services, experiences of care, and the acceptability of changes to workforce organisation and service delivery from a service‐user perspective. In publicly funded health and care systems, the public also has a legitimate stake in how the workforce is organised, supported, and sustained. This aligns with work on patient and public participation in health service governance and health policy decision‐making [8, 9]. Consistent with this broader literature, research across health research, including basic science, has reported unanticipated benefits from involving patients and public, such as more critical reflection on research priorities and the allocation and use of scarce resources [4].

Rather than questioning the legitimacy of public involvement, we argue for greater clarity, flexibility, and, where appropriate, expansion in how it is conceptualised and operationalised in workforce research. Re‐considering who counts as the ‘public’ in workforce research, and being transparent about the purposes and expected contributions of their involvement, is crucial to ensuring public involvement activities remain meaningful, proportionate, and fit‐for‐purpose when conducting workforce research. The moral and ethical principle underpinning public involvement—that research should be conducted ‘with’ or ‘by’ patients or members of the public, rather than ‘about’ them—applies equally to the health and care workforce, particularly as funders such as the NIHR now support an expanding portfolio of workforce research [2]. Yet workforce involvement is not currently treated as a mandatory and resourced component of research, creating a similar risk where studies are designed ‘about’ the workforce rather than shaped ‘with’ and ‘by’ them.

In practice, a broader definition of public involvement that encompasses ‘people’, rather than strictly patients and public, would enable involvement approaches that recognise the lived experiences of frontline staff most directly affected by the research topics, alongside professional and organisational representation within stakeholder engagement activities. Lessons may also be drawn from the more flexible ‘community engagement and involvement’ approach in NIHR's global health research portfolio, which emphasise context‐sensitive engagement, respect for individuals and communities, relationship‐building and trust, reciprocity, and partnership with diverse voices [10]; and similar principles in the Good Participatory Practice in clinical trials for engaging with the communities including the workforce itself [11]. Such flexibility is particularly relevant where the workforce under study extends beyond statutory services to include those in voluntary, community, faith and social enterprise sectors, as well as unpaid carers.

Elements of this broader approach are already evident in some projects, for example, through the use of professional or workforce panels and networks [12, 13] alongside dedicated patient and public involvement, allowing different forms of lived experience to contribute to different aspects of research. The UK‐REACH programme, a large interdisciplinary study of COVID‐19 outcomes among healthcare workers, for instance, established a professional and patient expert panel that brings workforce and public perspectives together in joint discussions, while also offering separate spaces where appropriate to support trust and openness [14]. Another approach involves engagement with patient and public representatives who hold governance or advisory roles within provider organisations and integrated care systems, who could bring system‐level insights into service delivery and workforce realities. Where appropriately designed, such arrangements may also enable public involvement resources to be used in ways that support meaningful inclusion of frontline staff with relevant lived experience, while helping to reduce practical barriers to participation for them.

However, any broadening of public involvement must remain attentive to issues of power and representation [15]. For example, combining frontline staff and public contributors in the same room may inhibit open discussion, not only because of formal hierarchies, but also staff may feel unable to speak candidly about everyday practice or system failures in front of patients and public contributors. Equally, there is a risk that expanding the definition of involvement in workforce research could be misinterpreted as a justification for displacing or downgrading patient and public perspectives altogether. Addressing this requires explicit processes for reflection, justification, equitable resource allocation, and accountability, ensuring that decisions about who is involved, how and why are transparent and aligned with the aims of the research, rather than driven by convenience.

As NIHR evolves its approach to public partnerships [16], there is an opportune moment to reflect on how public involvement is conceptualised and operationalised in workforce research. The ethical, moral, and methodological goals of public involvement remain valid and important for health and care workforce research. However, workforce research exposes tensions in how involvement and engagement are currently applied. Greater flexibility and reflexivity may help avoid tokenism, inefficiency, and misalignment between research purpose and involvement activities, and better recognise the role of frontline staff, patients and the public, and other stakeholders. Rather than offering definitive solutions, this viewpoint aims to open space for debate about how public involvement can be better aligned with the purpose and beneficiaries of workforce research, and call for future empirical studies examining the design, implementation, and impact of involvement in workforce‐focused research.

## Author Contributions

**Yingxi Zhao:** conceptualisation, project administration, visualisation, writing – original draft. **Justin Aunger:** conceptualisation, writing – review and editing. **Hilary Garrett:** conceptualisation, writing – review and editing. **Paul Hewitson:** conceptualisation, writing – review and editing. **Alun Davies:** conceptualisation, writing – review and editing. **Manish Pareek:** conceptualisation, writing – review and editing. **Sassy Molyneux:** conceptualisation, writing – review and editing.

## Ethics Statement

The authors have nothing to report.

## Conflicts of Interest

HG is a Public Advisory Co‐Lead with NIHR ARC NW Coast (Equitable Place‐Based Health and Care Theme); a public member of the NIHR Health and Care Professionals Doctoral Committee; a public contributor to the Models of Care Workstream for the Cross‐NIHR Collaboration on Multiple Long‐Term Conditions; a public member of the HDR UK North Executive Steering Board; and a Public Advisor (co‐production) for Lancashire County Council Adult Services Policy & Strategic Commissioning and the Social Care Institute for Excellence. PH is a Research Advisor and PPIE Officer for the Research Support Service at the University of Southampton and Partners. All other authors declare no conflict of interest.

## Data Availability

The authors have nothing to report.
